# Chronic Exposure to Cigarette Smoke Affects the Ileum and Colon of Guinea Pigs Differently. Relaxin (RLX-2, Serelaxin) Prevents Most Local Damage

**DOI:** 10.3389/fphar.2021.804623

**Published:** 2022-01-13

**Authors:** Chiara Traini, Silvia Nistri, Laura Calosi, Maria Giuliana Vannucchi

**Affiliations:** Department of Experimental and Clinical Medicine, Research Unit of Histology and Embryology, University of Florence, Florence, Italy

**Keywords:** mucins, blood vessels, inflammation, substance P, vasoactive intestinal peptide, mast cells, relaxin hormone

## Abstract

Cigarette smoking (CS) is the cause of several organ and apparatus diseases. The effects of smoke in the gut are partially known. Accumulating evidence has shown a relationship between smoking and inflammatory bowel disease, prompting us to investigate the mechanisms of action of smoking in animal models. Despite the role played by neuropeptides in gut inflammation, there are no reports on their role in animal models of smoking exposure. The hormone relaxin has shown anti-inflammatory properties in the intestine, and it might represent a putative therapy to prevent gut damage caused by smoking. Presently, we investigate the effects of chronic smoke exposure on inflammation, mucosal secretion, and vasoactive intestinal peptide (VIP) and substance P (SP) expressions in the ileum and colon of guinea pigs. We also verify the ability of relaxin to counter the smoke-induced effects. Smoke impacted plasma carbon monoxide (CO). In the ileum, it induced inflammatory infiltrates, fibrosis, and acidic mucin production; reduced the blood vessel area; decreased *c*-kit-positive mast cells and VIP-positive neurons; and increased the SP-positive nerve fibers. In the colon, it reduced the blood vessel area and the goblet cell area and decreased *c*-kit-positive mast cells, VIP-positive neurons, and SP-positive nerve fibers. Relaxin prevented most of the smoking-induced changes in the ileum, while it was less effective in the colon. This study shows the diverse sensitivity to CS between the ileum and the colon and demonstrates that both VIP and SP are affected by smoking. The efficacy of relaxin proposes this hormone as a potential anti-inflammatory therapeutic to counteract gut damage in humans affected by inflammatory bowel diseases.

## Introduction

Thirty years of scientific research have proven cigarette smoking (CS) as the main cause of lung cancer (Schwartz and Cote, 2016), as a promoter of pathologies of the respiratory and cardiovascular systems (Rigotti and Clair, 2013) and as one of the major risk factors for neuro-inflammatory and neurovascular disorders by supporting oxidative stress and inflammation (Aseervatham et al., 2017). Furthermore, CS is involved in several malignancies and intestinal inflammatory disorders such as Crohn’s disease (CD) (Verschuere et al., 2012a).

Nevertheless, investigation on the role played by CS in the gastrointestinal (GI) tract is limited. The experimental data available are often hardly comparable, mainly because of the different modalities of smoke exposure applied (active/passive smoking), the different smoke components studied [nicotine, carbon monoxide (CO), or whole cigarette], and the diverse administration methods used (oral, intraperitoneal, or subcutaneous). However, a region-specific modulation of the gut immune system has been reported after exposure to smoke. In the murine ileum, chronic smoke exposure caused an increase in apoptosis of the follicle-associated epithelium (Verschuere et al., 2012b; Allais et al., 2016; Allais et al., 2017), an increase in pro-inflammatory cytokines (Eliakim and Karmeli, 2003), and an excessive nitric oxide (NO) production (Allais et al., 2017). At variance with the ileum, the colon seems less sensitive to smoke irritants, as demonstrated by the failure of inflammatory cell recruitment in rats (Eliakim and Karmeli, 2003), the reduction of pro-inflammatory cytokines (Benson and Shepherd, 2011; Allais et al., 2017), and the changes in the CD4/CD8 ratio in mice (Daniluk et al., 2017).

This dual pattern of the responses of the small and large bowels to smoke was also observed in animal models of colitis (Galeazzi et al., 1999; Eliakim et al., 2001; Guo et al., 2001; Benson and Shepherd, 2011) and in human inflammatory bowel diseases (IBDs) (Berkowitz et al., 2018; Ananthakrishnan, 2013; Ananthakrishnan, 2015; Papoutsopoulou et al., 2020).

Interestingly, although vasoactive intestinal peptide (VIP) and substance P (SP) play a major role in the modulation of intestinal inflammation (Collins et al., 1999; Margolis and Gershon, 2009; El-Salhy et al., 2017), these two neuropeptides have never been studied in animal models of smoke exposure.

In this background, the hormone relaxin (RLX) emerges as an interesting putative therapy to preventing intestinal alterations caused by exposure to smoke. In fact, the human relaxin-2 (RLX-2) or serelaxin (Hossain and Wade, 2014) has shown anti-inflammatory and anti-apoptotic properties in both *in vitro* and *in vivo* animal models (Samuel et al., 2007). Moreover, RLX-2 has been shown to be protective in a guinea pig model of chronic exposure to CS, limiting vascular damage, lung inflammation, and fibrosis (Pini et al., 2016a; Pini et al., 2016b). In the gut, *in vitro* exposure to porcine RLX and *in vivo* porcine RLX chronic treatment caused muscle relaxation due to a region-specific modulation of the expressions of different NO synthase isoforms (Baccari et al., 2007; Vannucchi et al., 2011; Baccari et al., 2012). No data are available on the effect of RLX in the gut exposed to smoking.

On these premises, we designed the present study to investigate the effects of chronic exposure of guinea pig to CS on inflammatory response, mucosal secretion, and VIP and SP expressions in the terminal ileum and ascending colon by means of morphological and biomolecular methodologies. Contemporarily and as a novelty, we evaluated whether RLX-2, delivered continuously by subcutaneous osmotic pumps, was able to counteract CS-induced tissue damage.

## Materials and Methods

### Reagents

Clinical grade recombinant human RLX-2 (serelaxin) was kindly provided by the RRCA Relaxin Foundation (Florence, Italy). Kentucky Reference Cigarettes 3R4F, each containing 11 mg of total particulate matter, 9.4 mg of tar, and 0.73 mg of nicotine, were obtained from the Kentucky Tobacco Research Council (Lexington, KY).

### Animal Exposure to Cigarette Smoke

Male Hartley albino guinea pigs, weighing 300–350 g, were used for the experiments (Harlan, Correzzana, Italy). The use of male guinea pigs only had the aim of avoiding any interference with the endogenous relaxin produced during the post-ovulatory phase in females. Animal handling and use complied with the European Community Guidelines for Animal Care (2010/63/EU) and were approved and authorized by the Italian Minister of Health (code: 646/2015). The animals were housed on a 12-h light/dark cycle under standardized conditions of temperature and humidity with free access to food and water. The experiments were planned so as to minimize pain and the number of animals used.

The animals were divided into the following experimental groups (*n* = 5/group): controls, untreated animals (group 1); CS group, animals exposed daily to CS for 8 weeks (group 2); and CS+RLX-2 group, animals exposed daily to CS for 8 weeks and treated with RLX-2 (1 μg/day, s.c.) (group 3).

RLX-2 was administered by continuous subcutaneous infusion using osmotic mini pumps (Alzet; DURECT Corporation, Cupertino, CA, USA). The pumps were implanted on the back upon anesthesia [i.p. injection of 100 mg/kg body weight (b.w.) ketamine hydrochloride and 15 mg/kg b.w. xylazine] 1 day before starting exposure to CS and filled to deliver a daily dose of 1 μg for the whole duration of CS exposure. The RLX-2 dose of 1 μg/day was chosen according to the literature (Baccari et al., 2007; Bonacchi et al., 2009; Baccari et al., 2012; Pini et al., 2016a; Pini et al., 2016b).

Animals were subjected to CS exposure in a smoke chamber in accordance with Das et al. (2012), with minor modifications. The smoke chamber (2.5 L) was a vacuum desiccator equipped with an open tube for cigarette positioning at one end and a vacuum-connected tube and stopcock at the opposite end. To each group of CS-exposed animals, five 3R4F reference cigarettes were administered daily. Each cigarette was fitted into the inlet tube and lit; then, a puff of CS was introduced into the chamber containing the animals by applying a mild suction of 4 cm water for 20 s. The animals were exposed to the accumulated smoke for a further 40 s, for a total duration of CS exposure of 60 s. After a pause of 60 s, during which the chamber was opened and ventilated with fresh air, a second puff was administered with the same procedure. The gap between each of the 5 cigarettes/day was 1 h. At the end of the treatment, the animals were anesthetized with an i.p. injection of ketamine hydrochloride (100 mg/kg b.w.) and xylazine (15 mg/kg b.w.) for blood sampling from the left ventricle and killed by decapitation for tissue sample collection.

### Detection of Free Carbon Monoxide in Plasma

Free CO was measured in the plasma of the animals in the different experimental groups as an index of the degree of exposure to CS (Chang et al., 2017). The amount of free CO in plasma was measured with a gaseous CO detector (RGA3, Reduction Gas Analyzer; SAES Getters, Milan, Italy) as previously described (Vreman et al., 1984). Measurements were obtained by comparing with a standard CO curve prepared immediately before analysis and expressed as parts per million (ppm).

### Determination of Serum RLX-2 Levels

Circulating RLX-2 levels were determined in guinea pig serum with enzyme-linked immunosorbent assay (R&D Systems, Minneapolis, MN, USA) according to the manufacturer’s instructions.

### Tissue Sampling

Full-thickness samples of the ileum and the ascending colon (starting 1 cm far from the ileo-cecal junction) were taken from each animal, fixed in 4% paraformaldehyde in 0.1 M phosphate-buffered saline (PBS, pH 7.4) overnight (ON) at 4°C, dehydrated in graded ethanol series (2 h in 50% ethanol, 2 h in 75% ethanol, ON in 96% ethanol, and 2 h in 100% ethanol), cleared in xylene (1 h), and embedded in paraffin. Histological transverse (full-thickness) sections 5 μm thick were cut using a rotary microtome (MR2, Boeckeler Instruments Inc., Tucson, AZ, USA) and collected on normal or positively charged slides, as appropriate.

After excision, some specimens were cleaned of digestive material, and whole-tissue laminae containing the submucosa plus the circular muscle layer were obtained. The laminae were fixed in 2% paraformaldehyde in 0.1 M PBS with 0.2% picric acid (Zamboni’s fixative) for 2 h at 4°C. Subsequently, they were washed 3 times for 10 min each with dimethyl sulfoxide (DMSO) and 3 times for 10 min each with 0.1 M PBS and then stored in an anti-freeze solution (30% of ethylene glycol and 30% of glycerol in 0.1 M PBS) at −20°C.

### Histology and Histochemistry

Full-thickness sections were deparaffinized in xylene (2 steps, 5 min each) and rehydrated in a descending ethanol series (100%, 96%, 75%, and 50% ethanol, 2 min each step) to distilled water (5 min). Some sections were stained with hematoxylin–eosin (H&E) to evaluate the tissue architecture and to quantify the mean blood vessel area. Other sections were stained with periodic acid–Schiff (PAS) or toluidine blue (TB) to evaluate the quantity and quality of mucous secretion, respectively. PAS staining was performed according to the manufacturer’s instructions (Bio-Optica, Milan, Italy). For TB staining, the sections were soaked for 10 min in pre-filtered 0.1% TB in 30% ethanol. Other sections were stained with van Gieson (VG) to assess the submucosal collagen component. The sections were soaked for 30 s in van Gieson’s dye (0.1% acidic fuchsine in saturated picric acid solution) added with 2% acetic acid, then for additional few seconds in 50% van Gieson’s dye in water. All the stained sections were washed in distilled water, dehydrated in an ascending ethanol series, and mounted in synthetic resin.

### Immunohistochemistry

The sections were deparaffinized and rehydrated as usual, boiled 10 min in sodium citrate buffer (10 mM, pH 6.0; Bio-Optica) for antigen retrieval, and cooled at room temperature (RT). The sections were then rinsed in 0.1 M PBS and blocked for 20 min at RT with 1.5% bovine serum albumin (BSA; Sigma-Aldrich, Milan, Italy) in 0.1 M PBS. The primary antibodies, diluted in 1.5% BSA in 0.1 M PBS, were applied ON at 4°C. The day after, the slides were washed in 0.1 M PBS and incubated for 2 h at RT in the dark with appropriate fluorochrome-conjugated (Alexa Fluor 488 or 568) secondary antibodies diluted 1:333 in 0.1 M PBS. Tissue sections were thoroughly washed in 0.1 M PBS and mounted in an aqueous medium (Permafluor mountant; Thermo Fisher Scientific, Rockford, IL, USA). The primary antibodies used were as follows: rabbit polyclonal anti-*c*-kit antibody diluted 1:300 (Dako, Glostrup, Denmark); rat monoclonal anti-SP antibody diluted 1:100 (Santa Cruz Biotechnology, Santa Cruz, CA, USA); and rabbit polyclonal anti-VIP antibody diluted 1:50 (Santa Cruz Biotechnology). The secondary antibodies used were goat anti-rabbit (Jackson ImmunoReasearch Laboratories, West Grove, PA, USA) and goat anti-rat (Invitrogen, San Diego, CA, USA). To exclude nonspecific immunofluorescence staining, negative controls were performed by omitting the primary antibody. Immune reaction was observed under an epifluorescence Zeiss Axioskop microscope (Mannheim, Germany) using 488 or 568 nm excitation wavelengths and reading the fluorescence emission at 519 nm for the green label and at 603 nm for the red label. The fluorescence images were captured using a Leica DFC310 FX 1.4-megapixel digital camera, equipped with the Leica software application suite LAS V3.8 (Leica Microsystems, Mannheim, Germany).

### Morphometric Analysis

#### Histology and Histochemistry

The plasma cells and eosinophils in the mucosa were counted in the entire transversal sections of the ileum and colon (3 sections/animal, 5 animals/group).

PAS- and TB-stained goblet cells in the ileum and PAS- and TB-stained structures in the colon were evaluated in the entire transverse sections (2 sections/animal, 5 animals/group). Digital images were acquired with a video camera-equipped microscope (Eclipse 200; Nikon Instruments, Tokyo, Japan) with ×10 objective, paying attention not to overlap the microscopic fields. Quantitation was made using ImageJ software (NIH, Bethesda, MD, USA), as described. In the colon, PAS staining was commuted in 8-bit gray (Gy) images; then, a threshold of 135 in the specific window of the ImageJ software grayscale was settled and the area of the staining was calculated. The TB-positive area in the colon was selected using the color threshold window of ImageJ software, setting the following values: hue, 170; saturation, 60; and brightness, 180. Then, the area was quantified.

The blood vessel area (in square micrometers) was quantified in the submucosa and mucosa of the ileum and colon. A series of images covering the entire section of the ileum or colon (1 section/animal, 5 animals/group) stained with H&E was acquired with a ×40 objective and the reconstruction of the entire section made. The area was calculated with ImageJ software (NIH), drawing a circular line corresponding to the endothelial lining of the blood vessels. Only the vessels with a well-defined histological structure and containing red blood cells were included.

Images of VG-stained whole sections of the submucosa of the ileum and colon were acquired with a ×20 objective (1 section/animal, 5 animals/group). Quantitation of the red stained area, corresponding to collagen fibers, was made with ImageJ software as described: the red areas in the images of the ileum and colon were selected using the color threshold window of ImageJ software, setting the following values: hue, 190; saturation, 50; and brightness, 200. Then, each selected area was quantified.

#### Immunohistochemistry

The numbers of *c-*kit-positive mast cells (MCs) in the mucosa and the VIP-positive neurons in the submucosa were counted in the entire transverse sections of the ileum and colon (3 sections/animal, 5 animals/group).

SP-positive structures were quantified in the mucosa and submucosa of the ileum and colon. Images covering the entire section (1 section/animal, 5 animals/group) were acquired with a ×40 objective, paying attention not to overlap the microscopic fields. The photos were commuted in 8-bit Gy images and analyzed using ImageJ software upon setting a proper threshold value to only include SP-positive structures. Thresholds of 29 for the ileum and 24 for the colon were settled and the integrated density above the two thresholds calculated.

#### Statistical Analysis

All quantitative analyses were performed double blind by two trained observers (CT and MGV) and the values averaged. The result of each quantitation was expressed as the mean value per experimental group ± SEM. Statistical differences between values were calculated using one-way ANOVA followed by Newman–Keuls posttest. A *p*-value ≤0.05 was considered significant.

## Results

### General Effects

Observation of the guinea pigs exposed to CS did not point out any general signs of suffering (hair loss, aggressive behavior, or social isolation). Moreover, the animals were weighed at the beginning (control group, 361.8 ± 11.09 g; CS group, 339.5 ± 4.99 g; and CS+PLX-2 group, 335.2 ± 5.58 g) and at the end (control group, 891.3 ± 38.40 g; CS group, 873.8 ± 46.37 g; and CS+RLX-2 group, 798.8 ± 20.62 g) of the exposition, and statistical evaluation of the data showed no significant difference (*p* > 0.10).

### CO Levels in the Plasma

To check the effectiveness of the animal model, the levels of free CO in plasma were measured as an index of exposure to CS. All the animals exposed to chronic CS, independently of the RLX-2 treatment, had significantly elevated CO levels (CS group, 37.9 ± 3.2 ppm; CS+RLX-2 group, 32.1 ± 3.1 ppm) compared with the control group (6.4 ± 0.8 ppm: *p* < 0.001 *versus* the other groups). The absence of any difference between the two CS-exposed groups suggests that all animals underwent the same degree of CS-induced toxicity.

### RLX-2 Levels in the Serum

The level of RLX-2 in serum evaluated at the end of the experiment was 30 ± 4.4 pg/ml upon 1-μg daily doses. The values measured in the control and in CS-exposed animals not given RLX-2 were below the detection threshold.

### Ileum

#### Histology and Histochemistry

**FIGURE 1:**
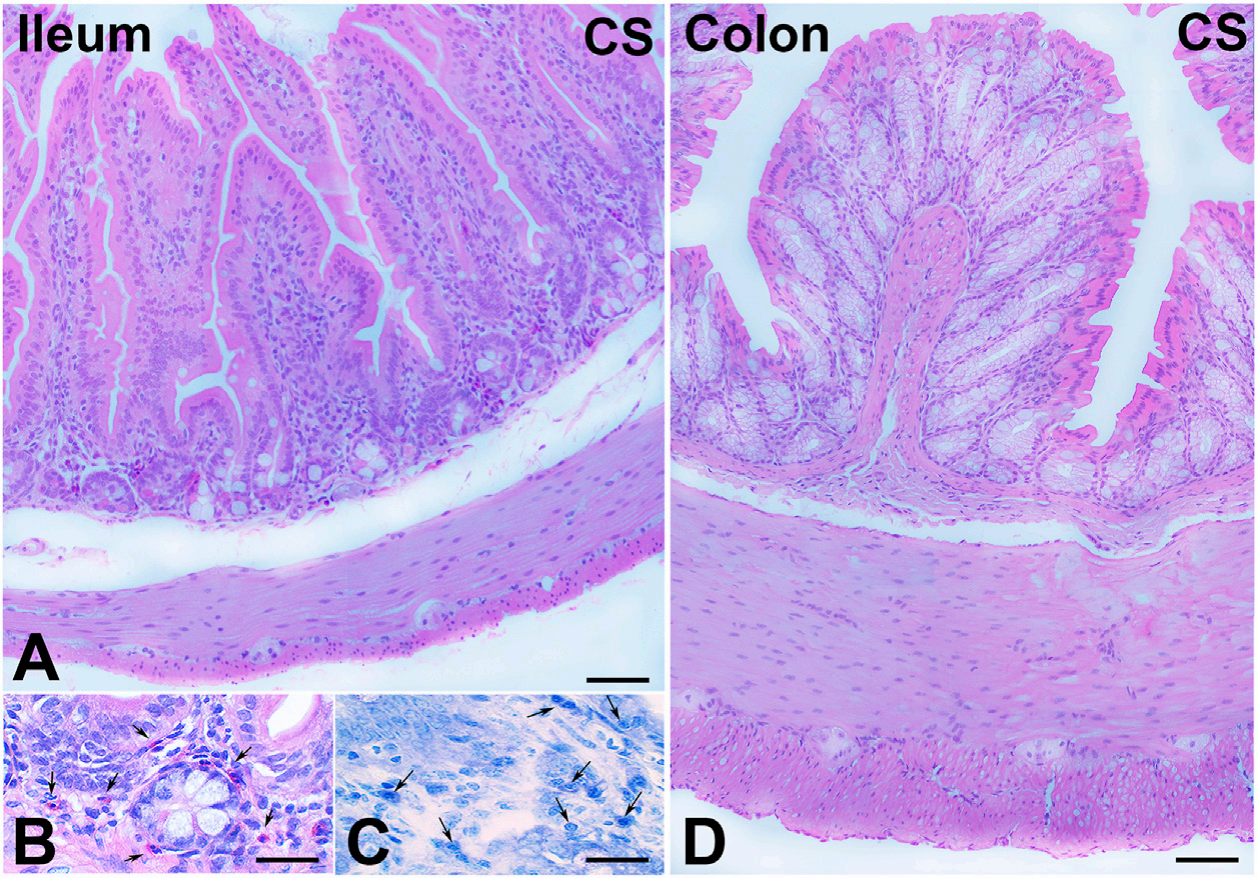
Hematoxylin–eosin (H&E) and Toluidine blue (TB) staining and cell infiltrates. **(A–C)** Ileum. **(A)** Full-thickness section showing the integrity of the enteric wall. **(B)** Numerous eosinophil granulocytes (*arrows*) in the mucosa. **(C)** TB staining pointing out several plasma cells (*arrows*) in the mucosa. **(D)** Colon. Full-thickness section showing the integrity of the enteric wall. *Bars*, 100 μm **(A,D)** and 25 μm **(B,C)**.

**FIGURE 2:**
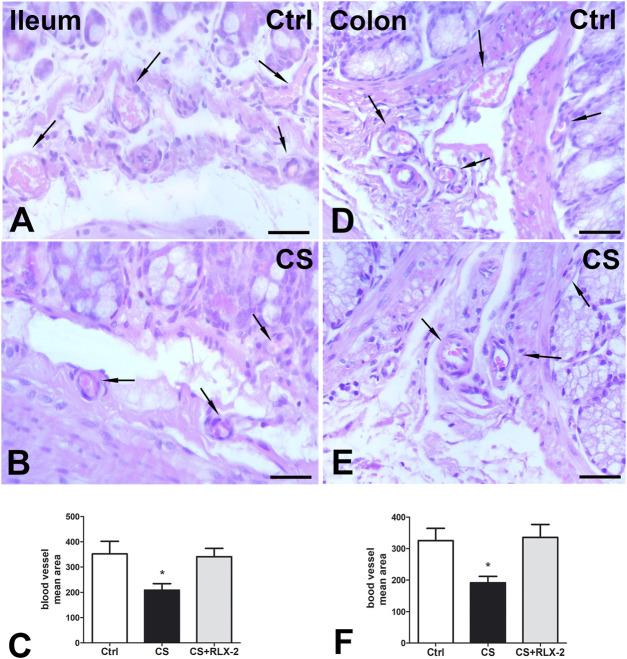
Hematoxylin–eosin (H&E) staining and blood vessel area changes. **(A,B)** Ileum. **(D,E)** Colon. Representative images of the submucosa and the mucosa of control **(A,D)** and cigarette smoke (CS)-exposed **(B, E)** guinea pigs showing several blood vessels containing erythrocytes (*arrows*). Quantitation of the blood vessel area in the ileum **(C)** and colon **(F)** demonstrated a significant decrease in CS-exposed animals. Relaxin-2 (RLX-2), chronically administered in CS-exposed animals, prevented this decrease **(C,F)**. *Bar*, 25 μm **(A,B,D,E)**. *p < 0.05.

H&E staining showed substantial integrity of the enteric wall in all groups of animals (Figure 1A). However, in the mucosa of the animals in the CS group, a slight inflammatory infiltrate, made up of plasma cells and eosinophilic granulocytes, was observed around the glandular fundus and in the surrounding connective tissue (Figures 1B, C). Quantitation of the plasma cells and eosinophils confirmed an increase in the CS group with respect to the control group; however, while the plasma cell counts gave a difference that did not reach statistical significance (*p* = 0.08; data not shown), the eosinophil counts reached significance: control group, 17.25 ± 2.7; CS group, 30.80 ± 4.1*; and CS+RLX group, 19.33 ± 3.1 (*p < 0.05). Numerous blood vessels were appreciable in the mucosa and submucosa (Figures 2A, B). Quantitative analysis of the mean blood vessel area revealed a significant reduction in animals in the CS group (208.8 ± 25.8 μm^2^) with respect to the controls (352.3 ± 49.9 μm^2^) and CS-exposed animals treated with RLX-2 (340.8 ± 33.0 μm^2^) (Figure 2C). Morphometric analysis of the red channel in the digital RGB images of VG staining showed a significant increase in the collagen fibers in the CS group compared with the other groups (Supplementary Figure S1).

**FIGURE 3:**
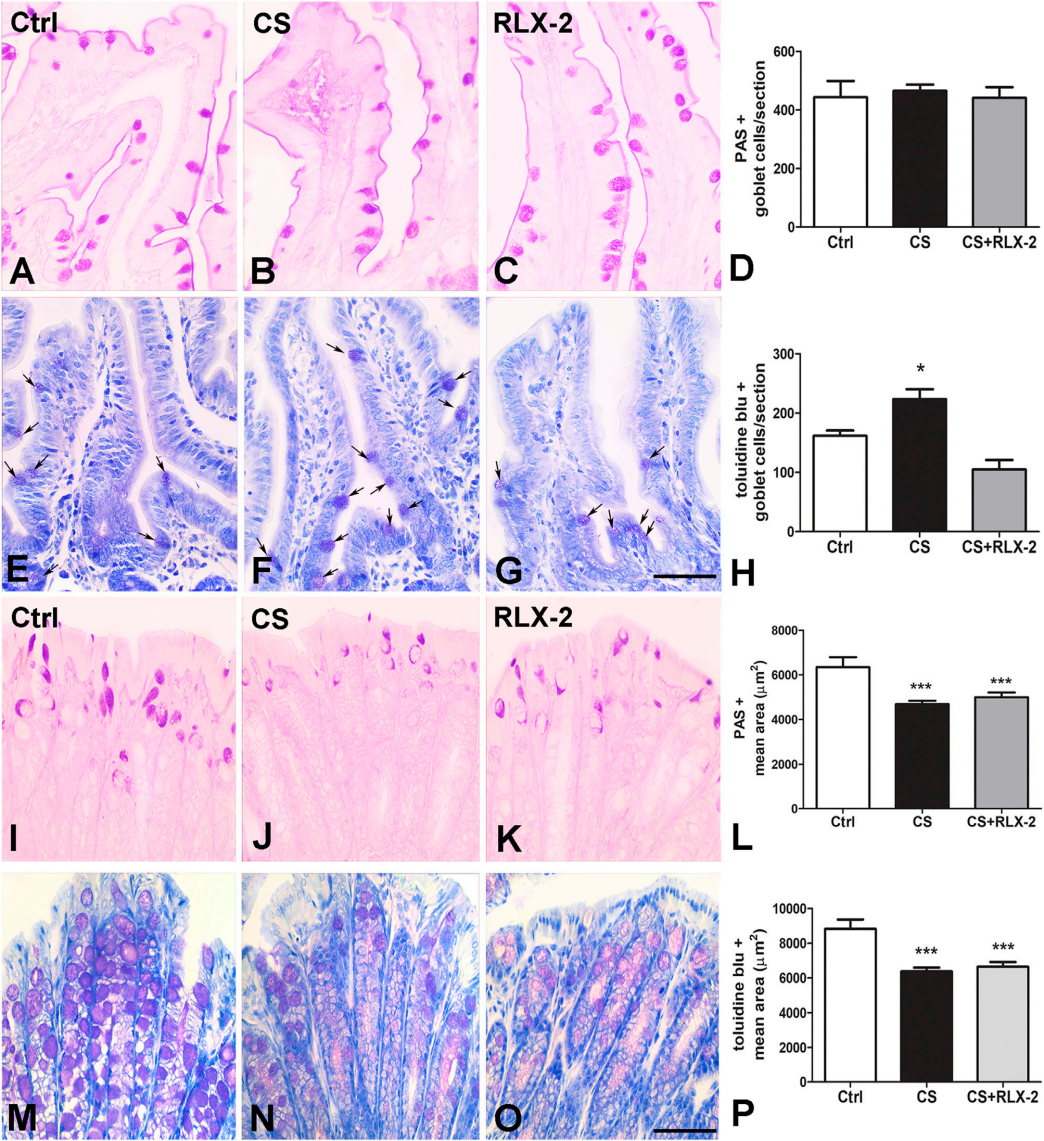
Periodic acid–Schiff (PAS) reaction and Toluidine blue (TB) staining and mucin changes in the cigarette smoke (CS) group. **(A–H)** Ileum. Representative images of the mucosa stained with PAS **(A–C)** and TB **(E–G)**. Both dyes stained the goblet cells. Quantitation of the stained cells showed no differences among the groups of animals using the PAS reaction **(D)**, whereas a significant increase in the number of goblet cells stained with TB was detected in CS-exposed animals **(H)**. This increase was prevented by relaxin-2 (RLX-2) treatment **(H)**. **(I–P)** Colon. Representative images of the mucosa stained with PAS **(I–K)** and TB **(M–O)**. Quantitation of the stained areas, with each dye showing a significant decrease in CS-exposed animals **(L**, **P)**. RLX-2 did not prevent the decreases **(L**, **P)**. *Bar*, 50 μm **(A**–**C**, **E**–**G**, **I**–**K**, **M**–**O)**. *p < 0.05; ***p < 0.0001.

The counts of PAS-positive goblet cells showed no differences among the experimental groups (control group, 443.0 ± 55.6; CS group, 465.0 ± 21.8; and CS+RLX, 441.1 ± 36.5) (Figures 3A–D). At variance, the number of cells stained with TB dye was significantly increased in CS-exposed animals (223.4 ± 16.8) with respect to control animals (161.5 ± 9.2) and CS-exposed animals treated with RLX-2 (104.8 ± 16.3) (Figures 3E–H).

#### Immunohistochemistry

##### c-Kit Immunoreactivity

**FIGURE 4:**
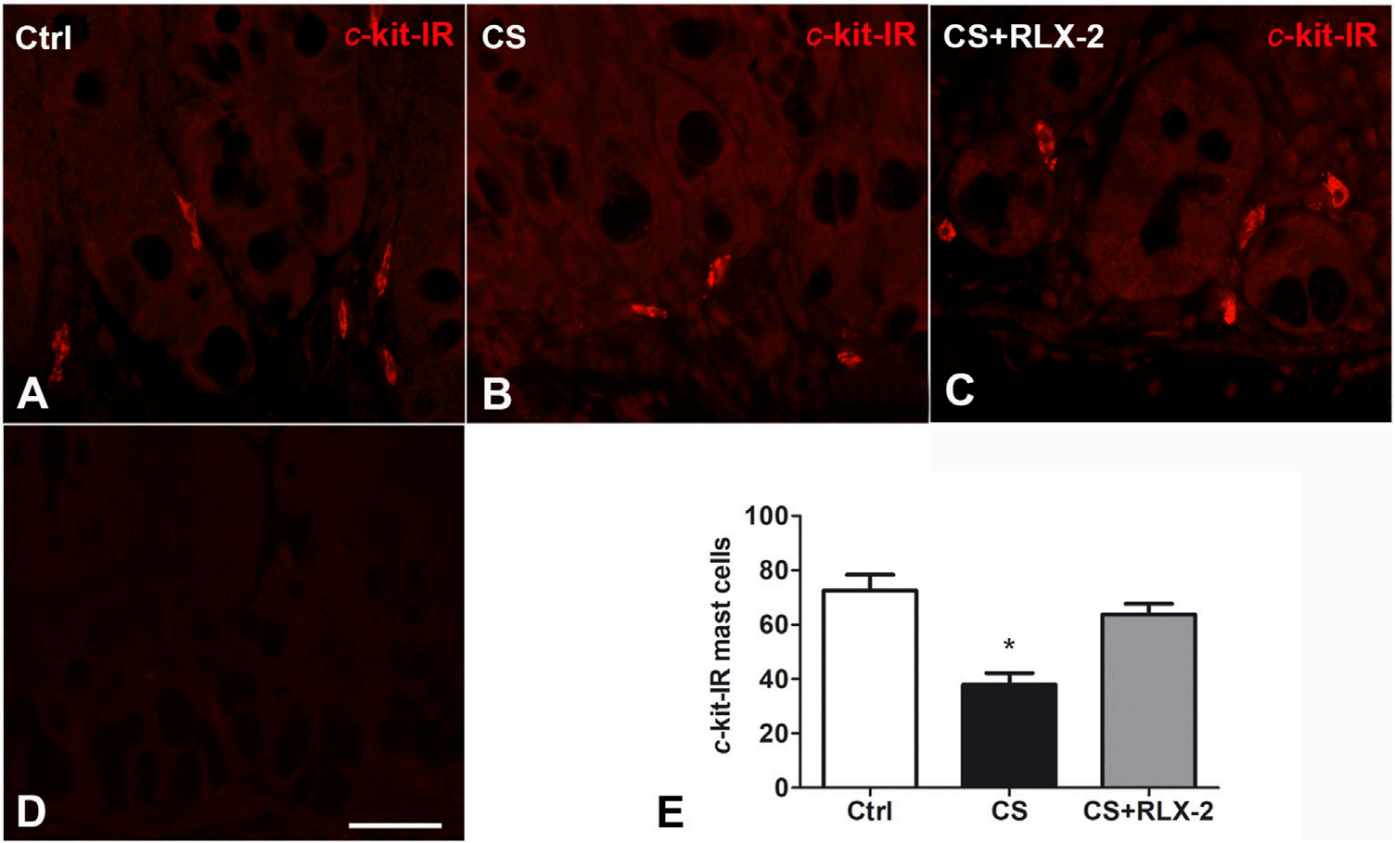
c-Kit-positive mast cells decreased in the cigarette smoke (CS) group. **(A–C)** Ileum. *c*-Kit-positive cells corresponding to mast cells are present in the mucosa surrounding the glandular fundus. **(E)** Quantitation of the number of *c*-kit-positive mast cells showed a significant decrease in CS-exposed animals. **(D)** Negative control. This decrease was prevented by relaxin-2 (RLX-2). *Bar*, 25 μm **(A–D)**. *p < 0.05.

MCs showing c-kit immunoreactivity (IR) were detected in the mucosa of all groups of animals (Figures 4A–C). Quantitation of the number of these cells showed a significant decrease in the CS group (38 ± 4.2) compared with control animals (72.7 ± 5.7) and CS-exposed animals treated with RLX-2 (63.82 ± 3.9) (Figure 4E). **Figure 4D** shows a mucosal image obtained in the absence of the primary antibody.

##### Vasoactive Intestinal Peptide Immunoreactivity

**FIGURE 5:**
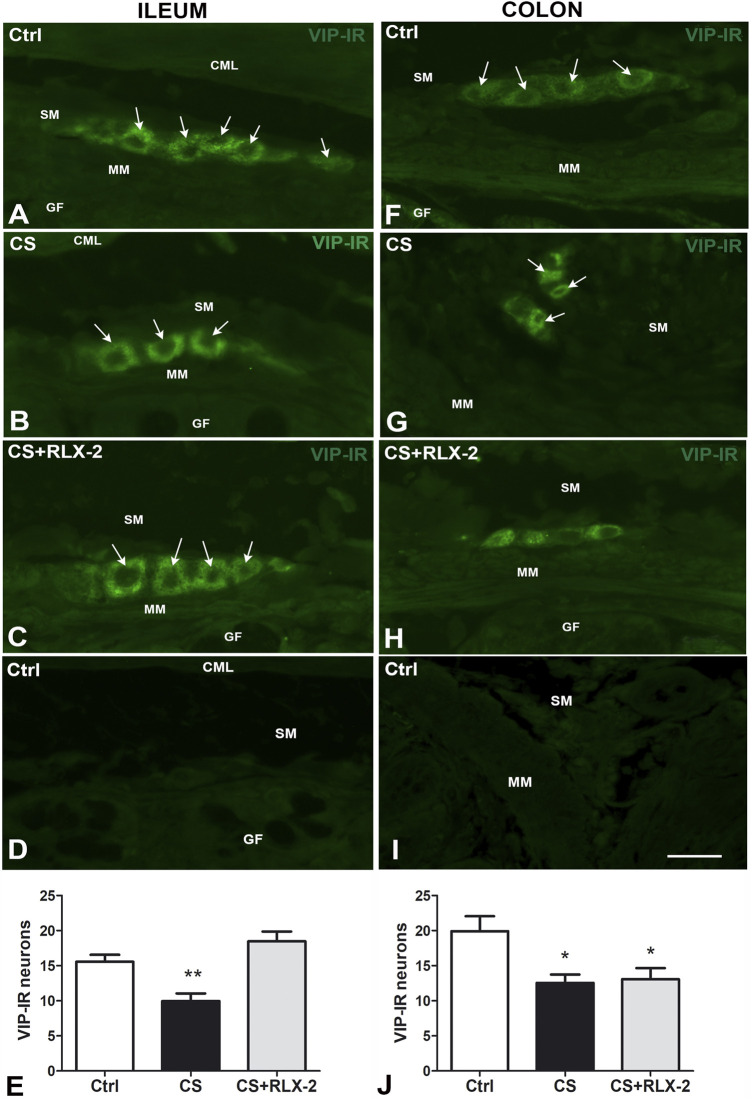
**A–I** Vasoactive intestinal peptide (VIP)-immunoreactive (IR) submucosal neurons in the ileum **(A–D)** and colon **(F–I)** of the cigarette smoke (CS) group and effects of relaxin-2 (RLX-2). Full-thickness sections. In both regions and in all groups of animals, labeling was detected only inside the ganglia. IR had a granular aspect and was located in the somata and in the first tracts of the processes. **(E)** In the ileum, the number of VIP-IR neurons was significantly decreased in CS-exposed animals, and RLX-2 administration prevented this decrease. **(D,I)**: negative controls. **(J)** In the colon, the number of VIP-IR neurons was significantly decreased in CS-exposed animals, but RLX-2 did not prevent this decrease. *CML*, circular muscle layer; *SM*, submucosa; *MM*, muscularis mucosae; *GF*, glandular fundus. *Bar*, 25 μm **(A–D; F–I)**. *p < 0.05; **p < 0.005.

In the submucosal plexus of the full-thickness sections, the VIP antibody labeled exclusively the neuronal cell bodies and, in some cases, the initial tract of the processes (Figures 5A–C). Quantitation of the VIP-IR neurons demonstrated a significant decrease in number in the CS group (9.9 ± 1.0) compared with the control group (15.6 ± 1.0) (Figure 5E, black and white columns, respectively). Chronic administration of RLX-2 in CS-exposed animals prevented this decrease (18.5 ± 1.4) (Figure 5E, gray column). Figure 5D is a submucosal image obtained in the absence of the primary antibody.

##### Substance P Immunoreactivity

**FIGURE 6:**
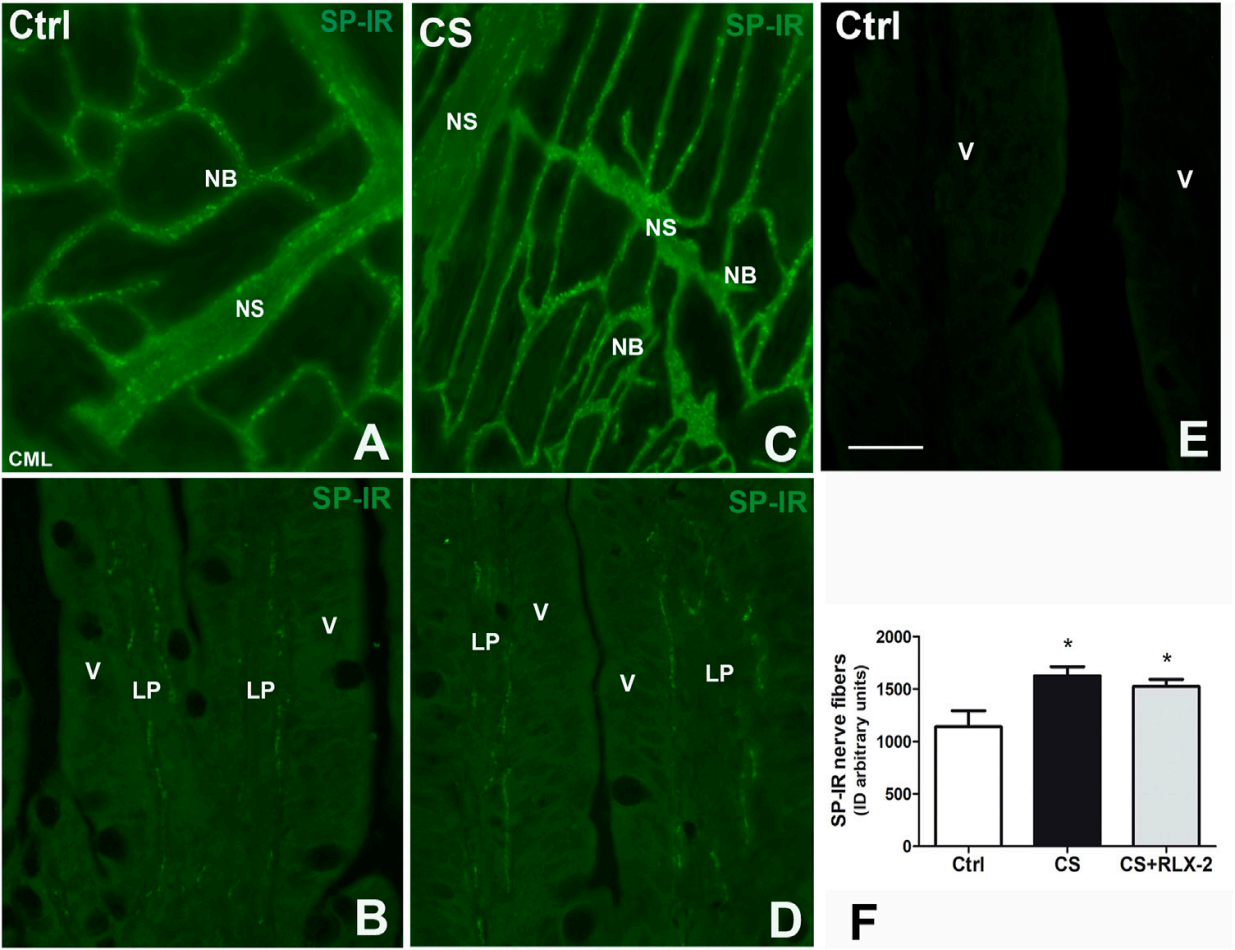
Increase in substance P (SP)-immunoreactive (IR) nerve fibers in the submucosa and the mucosa in the ileum of the cigarette smoke (CS) group and effects of relaxin-2 (RLX-2). **(A, C)** Laminae, submucosa. SP-IR was located in numerous nerve fibers forming nerve bundles (*NB*) and in the nerve strands (*NS*). **(B–E)** Full-thickness section, mucosa. Thin SP-IR nerve fibers ran along the axes of the villi (*V*) mostly lining the epithelium (*E*). **(E)** Negative control. **(F)** SP-IR nerve fibers significantly increased in the mucosa and submucosa of CS-exposed animals. This increase was insensitive to RLX-2 treatment. *CML*, circular muscle layer; *LP*, lamina propria. *Bar*, 50 μm **(A**–**E)**. *p < 0.05.

In the submucosal laminae, SP-IR was detected in numerous nerve fibers located along the nerve strands, in the nerve bundles (Figures 6A, C), and in the ganglia, surrounding negative neuronal bodies (data not shown). In the mucosa of full-thickness sections, the SR-IR nerve fibers followed the villus profile (Figures 6B, D). The labeling was granular, likely corresponding to the nerve varicosities (Figure 6). Quantitative analysis showed a significant increase in SP-IR nerve fibers, expressed as integrate density, in the CS group (1,626 ± 86.3) with respect to the control group (1,140 ± 152.3) both in the mucosa and submucosa (Figure 6F, black and white columns, respectively). RLX-2 was ineffective in preventing this increase in CS-exposed animals (1,524 ± 68.9) (Figure 6F, gray column). Figure 6E shows a mucosal image obtained in the absence of the primary antibody.

### Colon

#### Histology and Histochemistry

H&E staining showed substantial integrity of the colonic wall in all groups of animals, and no relevant signs of inflammation were visible (Figure 1D). As reported in the ileum, quantitative analysis of the mean vessel area in the colonic mucosa and submucosa showed a significant reduction in the CS group (191.6 ± 20.0 μm^2^) compared with the control group (325.4 ± 39.0 μm^2^) (Figures 2D–F), and RLX-2 prevented this reduction (335.5 ± 40.9 μm^2^) (Figure 2F). Morphometric analysis of the red channel in the digital RGB images of VG staining showed no differences among the groups (Supplementary Figure S1).

Quantitation of the PAS-positive (Figures 3I–K) and TB-stained (**Figures 3M**–**O**) areas showed a significant decrease in the CS group (4,689 ± 154.8 and 6,371 ± 213.4 μm^2^) compared with the control group (6,347 ± 441.7 and 8,817 ± 548.9 μm^2^) and the CS+RLX group (5,003 ± 209.4 and 6,635 ± 280.1 μm^2^) (Figures 3L, P).

#### Immunohistochemistry

The counts of MCs with c-kit IR showed a significant decrease in the mucosa of the CS group compared with the control group; this reduction was prevented by RLX-2 treatment (control group, 60 ± 4; CS group, 27 ± 3.4; and CS+RLX group, 44.4 ± 6.4).

VIP-IR was detected in the neuronal bodies of the submucosal ganglia only (Figures 5F–H), where it showed a granular pattern. Quantitation of the number of VIP-IR neurons showed a significant decrease in the CS group (12.6 ± 1.2) compared with the control group (19.9 ± 2.1) (Figure 5J, black and white columns, respectively). RLX-2 was ineffective in preventing this reduction (13.1 ± 1.6) (Figure 5J, gray column). Figure 5I is a submucosal image obtained in the absence of the primary antibody.

**FIGURE 7:**
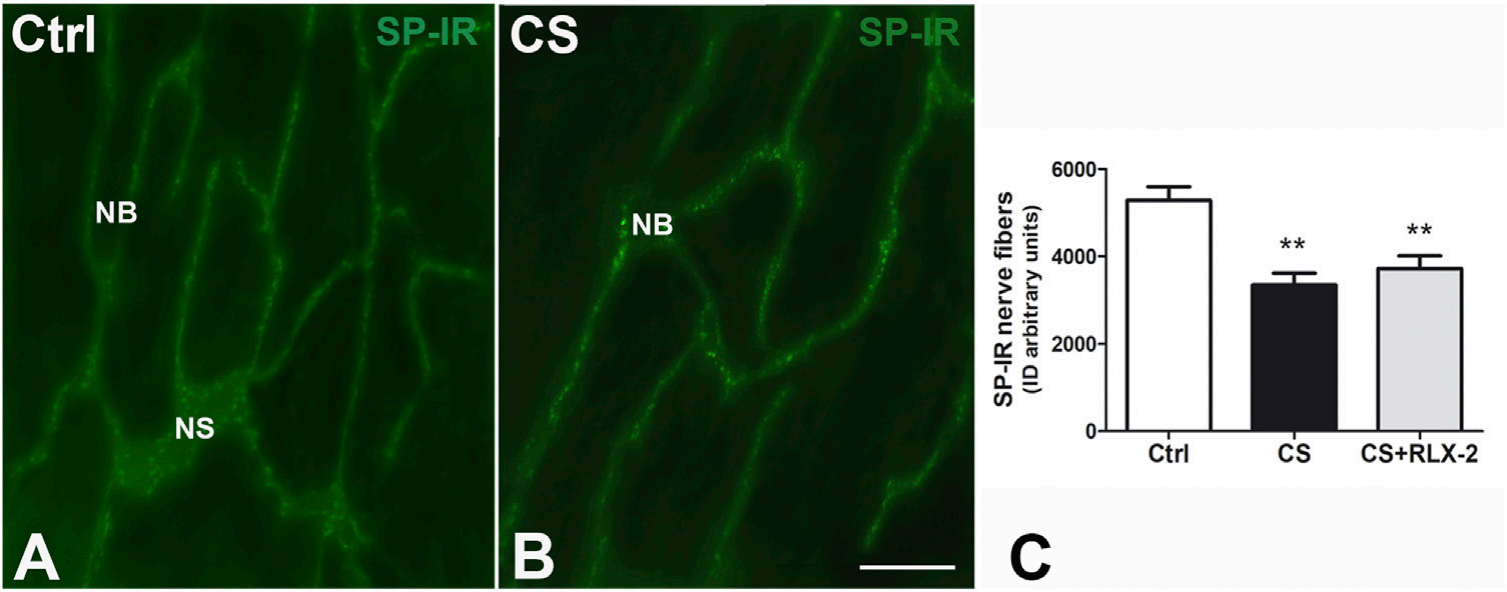
Decrease in substance P (SP)-immunoreactive (IR) nerve fibers in the submucosa in the colon of the cigarette smoke (CS) group and effects of relaxin-2 (RLX-2). **(A,B)** Laminae, submucosa. SP-IR was located in numerous nerve fibers that formed nerve bundles (*NB*) and in the nerve strands (*NS*). **(C)** The SP-IR nerve fibers significantly increased in CS-exposed animals. This increase was insensitive to RLX-2 treatment. *Bar*, 50 μm **(A**,**B)**. **p < 0.005.

SP-IR was identified in the nerve fibers of the submucosa and mucosa and has a punctate distribution**.** In the submucosal laminae, SP-IR was detected in the nerve fibers located along the nerve strands and nerve bundles (Figures 7A, B) and in the ganglia, surrounding negative neuronal bodies (data not shown). In the mucosa, SP-IR nerve fibers were few, scattered and located close to the epithelium (data not shown). Quantitation of SP-positive nerve fibers, expressed as integrate density, showed a significant decrease in the CS group (3,342 ± 274.7) with respect to the control group (5,285 ± 310.3), both in the mucosa and submucosa (Figure 7C, black and white columns, respectively). RLX-2 was ineffective in preventing this decrease (3,716 ± 298.1) (Figure 7C, gray column).

## Discussion

The present study demonstrated that chronic exposure to CS impacts on many of the investigated parameters, both in the ileum and in the colon. As a novelty, this study showed that chronic RLX-2 treatment prevented most CS-induced changes in the ileum, while it was less effective in the colon.

Mild inflammatory infiltrates, consisting mainly of acidophilic granulocytes and plasma cells, moderate degree of fibrosis, an increase in acidic muciparous goblet cells, vasoconstriction, a decrease in *c*-kit-positive MCs, VIP-positive neurons, and an increase in SP-positive fibers, were all detected in the ileum of CS-exposed guinea pigs. RLX-2, at a dose of 1 μg, was capable of preventing all these changes, except for the increase in SP-IR.

In the colon, CS induced vasoconstriction and significant reductions in mucin content, *c*-kit-positive MCs, SP-positive nerve fibers, and VIP-positive neurons. Unlike that in the ileum, in the colon, 1 μg RLX-2 only prevented vasoconstriction and reduction in MCs.

CS is the main cause of several pathologies (Rigotti and Clair, 2013; Schwartz and Cote, 2016; Aseervatham et al., 2017), and numerous molecules present in cigarettes or generated by their combustion produce cell damage through different mechanisms. Over the past decade, evidence of a close relationship between CS and IBDs has accumulated, prompting us to investigate the mechanisms of action of smoking by means of animal models. Due to the wide variety of these models and their results, we limited the discussion to those studies performed with models comparable to ours.

In general, animals chronically exposed to CS exhibited a more intense inflammatory reaction in the small intestine than in the large intestine (Verschuere et al., 2011; Wang et al., 2012; Zuo et al., 2014; Allais et al., 2016; Allais et al., 2017; Liu et al., 2020). Accordingly, we observed cell infiltrates and low-grade fibrosis in the ileum, but not in the colon. In both regions, however, we found a decrease in *c*-kit-positive MCs compared with the control specimens and a significant reduction in the blood vessel diameter. A reduced number of *c*-kit-positive MCs has been reported in bowel inflammatory disease and interpreted as a self-defense mechanism (internalization of the *c*-kit receptor) in the presence of noxious stimuli (Farhadi et al., 2007). Therefore, in our experimental conditions, the reduced expression of the surface *c*-kit receptor in MCs might have been dependent on the irritative activity of CS. We might wonder whether a decreased expression of *c*-kit labeling also corresponds to loss of MCs.

Based on the observations that, in the guinea pig lung, CS caused MC degranulation (Pini et al., 2016a) with an increase in vascular resistance and permeability (Sun et al., 2021), we interpreted the reduced number of *c*-kit-positive MCs as the consequence of cell degranulation rather than an actual loss. In favor of this possibility are the presence of vasoconstriction and the effects of RLX-2. In fact, in the guinea pigs exposed to CS and treated with RLX-2, the number of *c*-kit-positive MCs and the caliber of the blood vessels were comparable to those of the control group, indicating that the two phenomena are related and likely dependent on the ability of RLX-2 to inhibit ileal MC degranulation. Indeed, several reports in tissues and organs (Masini et al., 1994; Masini et al., 1995; Nistri et al., 2008; Pini et al., 2016a) have proven that RLX-2 prevents the degranulation of MCs. Finally, the maintenance of the number of MCs in RLX-2-treated CS-exposed guinea pigs supported the above-mentioned interpretation.

Chronic exposure to CS also caused changes in mucosal secretion. The capacity of CS to alter mucin production was reported in human lungs affected by chronic obstructive pulmonary disease (COPD) (Di et al., 2012; Kim, 2012) and in guinea pig airways (Pini et al., 2016b). The important role of mucins in maintaining intestinal homeostasis has been amply described (Snyder and Walker, 1987; Rhodes, 1997; Liu et al., 2020). Changes in the composition or quantity of mucins are common in inflammatory and neoplastic gut diseases (Rhodes, 1997; Shirazi et al., 2000; Al-Khayal et al., 2016; Allais et al., 2016), as well as in conditions of dysbiosis (alteration of the microbiota). Very few data are available on the effects of CS on mucin production in the gut. In mice exposed for 24 weeks to CS, Allais et al. (2016) showed an increase in the expression of mRNA encoding for secretory MUC2 and cell surface MUC3 in the ileum, as well as for cell surface MUC4 in the distal colon, without changes in the quantity and quality of goblet cells. At variance with these findings, we observed a significant shift toward an acidification of mucins in the ileum and a significant decrease in mucin content in the colon. The discrepancies between the results of Allais and coworkers and our results are likely related to differences in the time of CS exposition and the animal species used. However, the diverse patterns of mucin secretion following CS exposure in the two intestinal regions were in agreement with literature data (Allais et al., 2016; Allais et al., 2017). Presently, the shift toward more sulfated mucins in the ileum was interpretable as an attempt at defense against CS-mediated mucosal aggression. In fact, sulfation increased the anionic charge of mucus, making the carbohydrate side chains more resistant to bacterial enzymes and boosting the resistance of the epithelium to microbial invasion (Rhodes, 1997). In the colon, instead, the decrease in mucin content might have been dependent on the CS-induced dysbiosis. In fact, Allais et al. (2016; 2017), in the colon of mice chronically exposed to CS, reported the presence of dysbiosis characterized by an impairment in mucin-producing bacteria. Accordingly, in mice exposed for 10 weeks to CS, Zuo et al. (2014) reported the presence of inflammation in the ileum, but not in the colon, and attributed these different CS effects to the intrinsic properties of the two enteric regions; among these properties, the richness and variety in colonic microbiota might have played a main role (Zuo et al., 2014). Interestingly, a different sensitivity to CS was also reported in humans for recurrent IBD, i.e., CD and ulcerative colitis (UC). In fact, CS exacerbated the clinical course of CD, whereas ex-smokers affected by UC showed an increased frequency of disease relapse or worsening (Ananthakrishnan, 2013; Ananthakrishnan, 2015; Allais et al., 2017).

RLX-2 administration to CS-exposed animals prevented the changes in mucin quality in the ileum, but had no effect in the colon. These results might be due to the different origins of mucin alterations in the two regions. In the ileum, where the mucin changes likely have an inflammatory origin, RLX-2 was effective because of its anti-inflammatory properties (Nistri et al., 2003; Nistri et al., 2008; Ng et al., 2019). Conversely, in the colon, where the decrease in mucin content was chiefly of bacterial origin (Allais et al., 2016), RLX-2 had no effect.

In the current study, we investigated, for the first time, the distribution of SP and VIP immunoreactivity in the ileum and colon of animals chronically exposed to CS. SP and VIP are the two main mediators for the local regulation of sensation, secretion, vasomotor, and neuro-inflammation. In a pathophysiological context, SP and VIP are considered as natural pro- and anti-inflammatory neuropeptides, respectively (Margolis and Gershon, 2009; Pelayo et al., 2014; Allais et al., 2017; El-Salhy et al., 2017; Patel et al., 2020). In the guinea pig ileum and colon, SP nerve fibers are mainly concentrated in the mucosa and submucosa (Portbury et al., 1996; Lomax et al., 1998; Harrington et al., 2005), while VIP-positive neurons are more numerous in the myenteric than in the submucosal plexus; in both plexuses, the majority of VIP-positive neurons also express neuronal nitric oxide synthase (nNOS) (Chino et al., 2002).

To date, the possibility that CS could affect intestinal neuropeptides has only been investigated in patients suffering from IBD or in IBD animal models. In CD and UD, a significant increase of SP expression in the mucosa and submucosa was described to be positively correlated with the degree of inflammation (Weinstock, 2015; Allais et al., 2017; Allais et al., 2020; Patel et al., 2020). However, divergent findings have also been reported (Margolis and Gershon, 2009). Studies on VIP expression in patients with IBD have produced even more conflicting results (Margolis and Gershon, 2009; El-Salhy et al., 2017). These apparently contradictory data conceivably are due to several reasons, i.e., the use of small, limited endoscopic specimens; the regions investigated, mainly the rectum and final tract of the distal colon; the concurrent drug therapy; and the stage of the disease (Margolis and Gershon, 2009; El-Salhy et al., 2017). On the contrary, the more standardized studies on IBD-like animal models have shown a constant increase in SP and a decrease in VIP expression during the active phases of the diseases (O’Connor et al., 2004; Gonzalez-Rey et al., 2006; Margolis and Gershon, 2009; El-Salhy et al., 2017). Taken together, these reports highlighted the close relationship between abnormalities in the neuronal expression of VIP and SP and mucosal dysfunction in the presence of chronic inflammation.

Under the present experimental conditions, CS significantly influenced the expressions of both neuropeptides with important differences between the ileum and colon. In the ileum, similarly to those reported in animal models of chronic inflammation, CS decreased VIP and increased SP expression. The SP increase might depend on the ability of CS to activate the extrinsic capsaicin-sensitive sensory neurons (Price and Flores, 2007; Allais et al., 2017), which, as reported (Allais et al., 2017; Allais et al., 2020), synthesized and released SP. This neuropeptide binds with high affinity to the NK1 receptor, which, in the ileal guinea pig mucosa, is expressed by secretomotor cholinergic neurons (Lomax et al., 1998) and by several immune cells (Margolis and Gershon, 2009; El-Salhy et al., 2017; Patel et al., 2020). An increased activation of these targets mediated by SP might generate/potentiate a local inflammatory reaction. The decrease of VIP neurons observed in our experiments is in agreement with literature data on IBD, although the involved mechanism remains undefined (O’Connor et al., 2004; Gonzalez-Rey et al., 2006; Margolis and Gershon, 2009; El-Salhy et al., 2017). The finding that RLX-2 treatment prevented the VIP decrease, but not the SP increase, suggests that the modifications of the two neuropeptides followed two parallel but distinct pathways. RLX has shown tissue protection, reducing the inflammatory infiltrates and tissue oxidative stress and regulating the endogenous production of nitric oxide through modulation of the different NO synthases (Masini et al., 1994; Masini et al., 1995; Nistri et al., 2008; Baccari et al., 2012; Pini et al., 2016a; Pini et al., 2016b; Bani, 2020). In the guinea pig submucosal ganglia, the vast majority of the VIP-IR neurons co-expressed nNOS, and at variance with the myenteric ones, none of them expressed tachykinin receptors (Portbury et al., 1996; Lomax et al., 1998; Harrington et al., 2005; Pelayo et al., 2014). This latter finding excludes that the SP increase might directly interact with the VIP/nNOS-IR neurons. On the contrary, the activation of MCs, as suggested by the present findings, and the recruitment of inflammatory cells able to produce NO at high concentrations (Eliakim et al., 2001; Verschuere et al., 2011; Wang et al., 2012; Zuo et al., 2014; Allais et al., 2016; Allais et al., 2017; Liu et al., 2020) might interfere with the activity of the VIP/nNOS-IR neurons (Colasanti and Suzuki, 2000; Traini et al., 2021). In this scenario, RLX-2, by preventing the activation of MCs and inhibiting the recruitment of inflammatory cells, would ensure the normal activity of VIP/nNOS-IR neurons, which in turn may potentiate the anti-inflammatory effects of RLX-2 (Margolis and Gershon, 2009; Pelayo et al., 2014:; El-Salhy et al., 2017; Allais et al., 2017; Patel et al., 2020).

In the colon of CS-exposed guinea pigs, the expressions of SP and VIP were significantly decreased. These results are quite surprising, unless framed in the general behavior of the colon in the presence of CS. As reported, Allais et al. (2016, 2017) found significant changes, in both quantity and quality, of the colonic microbiome upon CS exposition, and they attributed this effect to the far higher bacterial load in the colon compared with that in the ileum. Among these microbiota changes, the authors also reported an increased activity of Lachnospiraceae sp., which are butyrate producers. Because of the anti-inflammatory properties of butyrate, an increase in its production might explain the scarce inflammatory reaction commonly observed in the colon compared with the ileum (Verschuere et al., 2012a; Verschuere et al., 2012b; Allais et al., 2016; Allais et al., 2017). Based on their own data and those from the literature, the authors concluded that the responses of the microbiota to CS are double-faced: if the loss of some species might compromise mucin production, thus lowering the mucosal barrier defense, the increase of butyrate-producing species hampers local inflammation and potentiates the epithelial barrier (Wang et al., 2012; Allais et al., 2016; Allais et al., 2017).

In summary, taking our data and those from the literature together, it might be hypothesized that the lower sensibility and the limited damage caused by CS in the colon depended on a downward readjustment of the organ functions and that the microbiota played a key role in this readjustment. Intriguing and significant are the data from the CS-exposed animals treated with RLX-2. Indeed, this hormone showed the ability to counteract almost all the changes caused by CS in the ileum, as well as the activation of MCs and the vasoconstriction in the colon. These newly identified properties of RLX-2 (available as a pharmaceutical form under the name serelaxin) extended its panel of protective effects on the intestine exposed to irritative noxae and suggested that it could be tested as a potential new anti-inflammatory therapeutic to counteract the gut damage in smokers and in those affected by IBD.

The present research, using mainly morphological techniques, has allowed demonstrating the presence of significant and never before reported findings, such as the involvement of submucosal neurotransmission following exposure to CS and the human hormone RLX-2, administered at physiological doses, protecting the intestinal mucosa from CS damage. It will be useful to complete the study by extending the investigation to the muscle wall and myenteric neurons. Indeed, although changes in gut motility are present in smokers and, mostly, in people affected by IBDs, this topic is very little investigated. In this regard, an interesting experimental paper has been recently published (Balasuriya et al., 2020). In perspective, we could apply our methodology to investigate whether RLX-2 is able to prevent the increase in cell infiltrates consisting of eosinophils and the decrease in SP and VIP expressions reported in the colon of animal models (Traini et al., 2017; Traini et al., 2021) that mimic the most common intestinal morpho-functional disease, irritable bowel syndrome.

## Data Availability

The original contributions presented in the study are included in the article/Supplementary Material, further inquiries can be directed to the corresponding author.
