# Supplementary figures and images for: Chronic Exposure to Cigarette Smoke Affects the Ileum and Colon of Guinea Pigs Differently. Relaxin (RLX-2, Serelaxin) Prevents Most Local Damage
Source: Front Pharmacol. 2022 Jan 13;12:804623. doi: 10.3389/fphar.2021.804623 (PMC8793690; doi:10.3389/fphar.2021.804623)

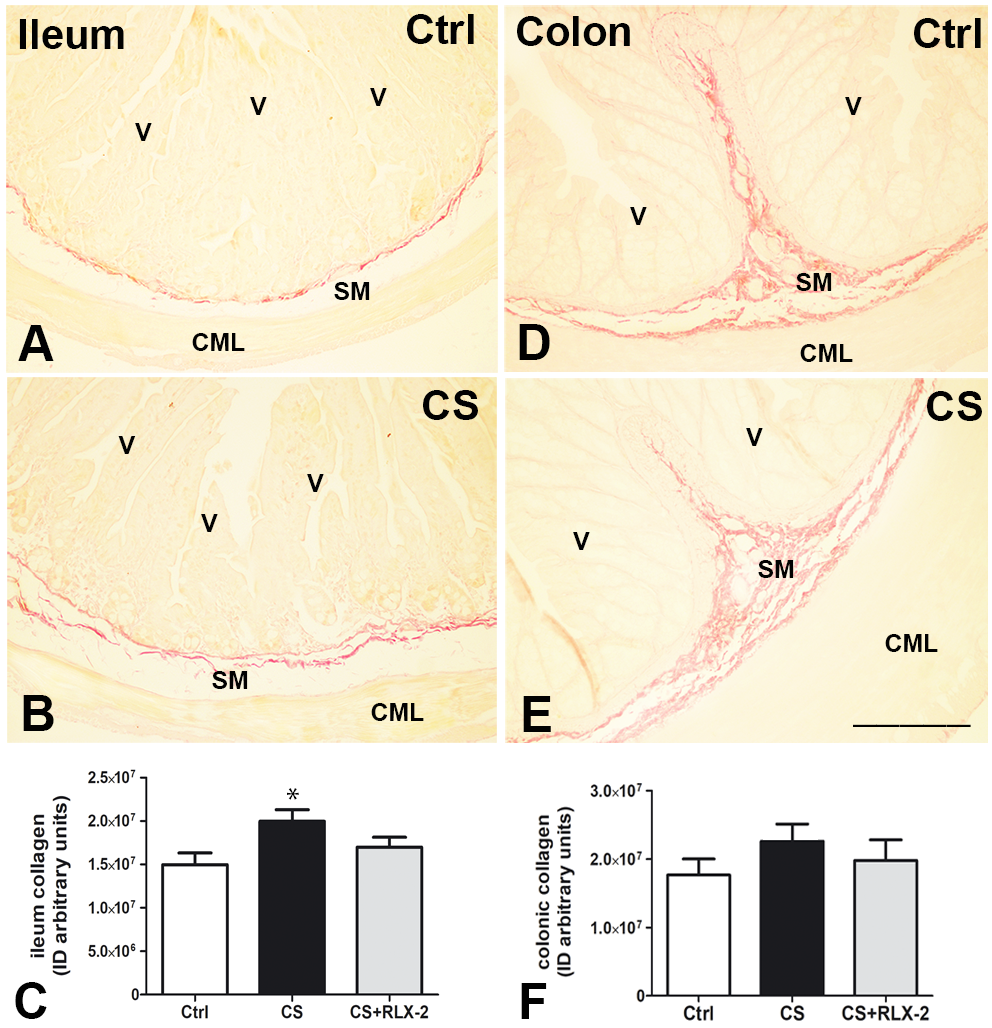

Supplement: Supplementary file 1 [file Image1.TIF]
